# Eruptive Syringoma—Clinical, Dermoscopic, and Reflectance Confocal Microscopy Features

**DOI:** 10.3390/diagnostics15010110

**Published:** 2025-01-04

**Authors:** Agnieszka Rydz, Jakub Żółkiewicz, Michał Kunc, Martyna Sławińska, Michał Sobjanek, Roman J. Nowicki, Magdalena Lange

**Affiliations:** 1 Student’s Scientific Circle Practical and Experimental Dermatology, Medical University of Gdańsk, 80-214 Gdańsk, Poland; 2 Department of Dermatology, Venereology and Allergology, Medical University of Gdańsk, 80-214 Gdańsk, Poland; 3 Department of Pathomorphology, Medical University of Gdańsk, 80-214 Gdańsk, Poland

**Keywords:** eruptive syringoma, syringoma, cutaneous mastocytosis, dermoscopy, dermatoscopy, reflectance confocal microscopy, histology

## Abstract

We present an interesting image of eruptive syringoma confirmed by histopathological assessment in a 37-year-old male who was consulted due to numerous brownish small macules and papules resembling maculopapular cutaneous mastocytosis (MPCM). We show difficulties in diagnosing ES, given its rare occurrence and resemblance to other dermatological disorders. Moreover, we discuss the role of dermoscopy and reflectance confocal microscopy in the differential diagnosis of syringoma.

**Figure 1:**
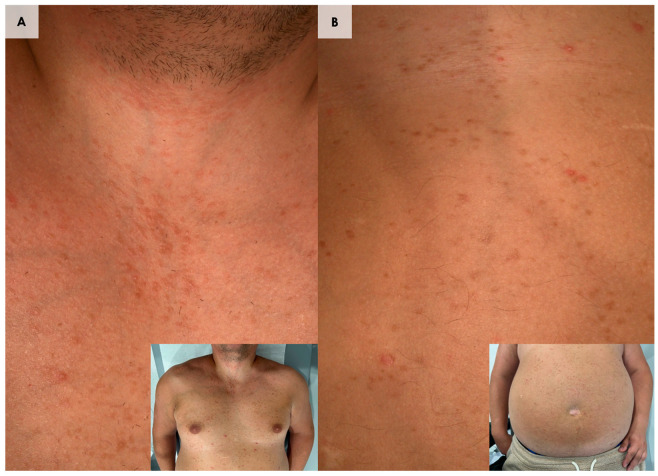
(**A**,**B**). We present a 37-year-old overweight male who presented with numerous small, monomorphic, brownish macules and papules scattered across the trunk, neck, and thighs (**A**,**B**). The patient exhibited multiple flat-topped, firm, hyperpigmented brown macules and papules, varying in size from 1 to 3 mm, distributed across the neck, chest, abdomen, back, and lower extremities. The lesions were predominantly located on the anterior aspect of the body. According to the patient, skin lesions have been present since elementary school and remained stable without progression to other areas of the body. The patient was referred to our department under suspicion for maculopapular cutaneous mastocytosis (MPCM). Nevertheless, Darier’s sign, which is pathognomonic for cutaneous mastocytosis, was negative [1]. Moreover, he had no cutaneous and systemic mast cell mediator-related symptoms typical for mastocytosis [1]. There was no history of an anaphylactic shock, allergic reactions, or a chronic illness. Complete blood count with differential, biochemistry, and serum tryptase level (3.35 ng/mL, range up to 11.4 ng/mL) were in normal ranges. Due to an unclear clinical presentation, dermoscopy (Figure 2A,B) and reflectance confocal microscopy (RCM) (Figure 3 and Figure 4) were performed. Two skin biopsies from the skin of the trunk were performed, and histopathological examination indicated a diagnosis of syringoma in both samples (Figure 5). Due to the disseminated distribution of skin lesions, a final diagnosis of eruptive syringoma (ES) was established. Our patient was informed that syringoma is a benign tumor that usually remains unchanged over time and does not pose significant health risks. Moreover, various treatment modalities were offered to the patient, including surgical removal, dermabrasion, electrocautery, cryosurgery, chemical peels, topical atropine, botulinum toxin A, and oral medications, such as isotretinoin [2,3]. However, the patient was not interested in treatment for solely aesthetic reasons. Syringoma is a benign skin neoplasm characterized by the overgrowth or hyperplasia of eccrine sweat ducts, leading to the formation of small, flat, skin-colored, or brownish papules [4,5]. Syringomas are typically located in the periorbital region; however, they have also been found in other locations, such as the trunk, extremities, vulva, penis, scalp, and underarms [6]. There are four variants of syringoma: the localized form, the familial form, a form associated with Down syndrome (DS), and the generalized variant [7]. Eruptive syringoma, a rare form of the generalized variant, is characterized by the sudden appearance of multiple lesions that spread across two or more anatomical regions [2]. The exact etiology of ES remains unclear, but it is believed to result from reactive hyperplasia of the eccrine ducts, potentially triggered by chronic inflammation, hormonal changes, or other unknown factors [8]. The condition is more prevalent in females and often manifests during puberty or adolescence, indicating a possible hormonal influence, but ES has also been reported in children and the elderly [2,9]. This case report indicates that ES poses a diagnostic challenge because its clinical presentation can be easily mistaken for other dermatological conditions, such as eruptive xanthomas, disseminated granuloma annulare, MPCM, lichen planus, flat warts, or eruptive vellus hair cysts [10]. Therefore, using dermoscopy and RCM may provide useful clues in the diagnostic process (Supplementary Table S1). However, histopathological examination remains the gold standard in the diagnosis of syringomas, as clinical presentation and non-invasive skin imaging techniques alone are not sufficient to distinguish ES from other skin lesions considered in the differential diagnosis.

**Figure 2:**
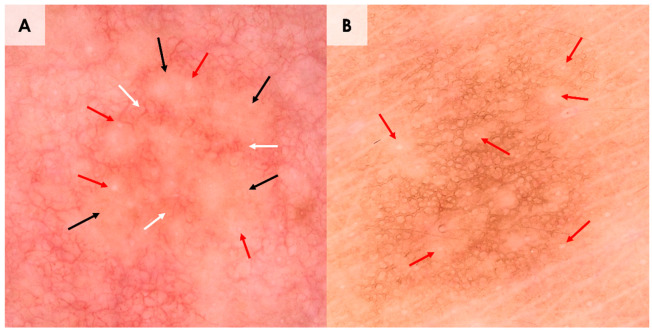
(**A**,**B**). Dermoscopy of syringomas located on the neck (**A**) and trunk (**B**). Dermoscopy of a syringoma located on the neck (**A**) showed linear vessels in reticular distribution (white arrows) and light-brown structureless areas (black arrows) with small whitish globules (red arrows). Dermoscopy of the lesion located on the trunk (**B**) revealed brown reticular lines (pigment network) and white dots/globules (red arrows). Dermoscopic manifestations of ES differed according to the anatomical region. In the neck area, vascular pattern was observed, whereas pigment network prevailed in the abdominal region. In both instances, white dots/globules scattered across the lesion were identified. A similar location-dependent dermoscopic presentation was described in a case report by Botsali et al. [11]. Pigment network was identified in syringoma cases reported by Sakiyama et al. and Hayashi et al. [12,13]. The latter group also described multifocal hypopigmented areas under dermoscopy, as described in the reported patient.

**Figure 3:**
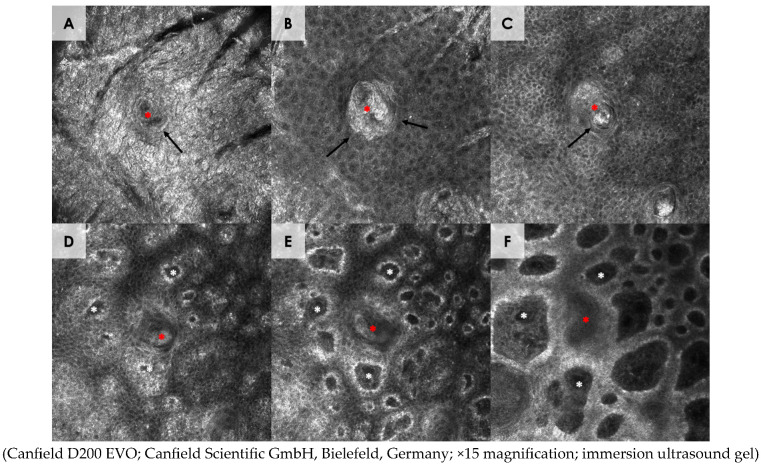
Reflectance confocal microscopy of syringoma. Dark hole (red asterisk) surrounded by bright, highly reflective layers of cells (black arrows), which correspond to acrosyringium, are seen within the stratum corneum (**A**), stratum granulosum (**B**), and stratum spinosum (**C**). A highly reflective layer, composed of densely packed, anucleated keratinocytes, constitutes the stratum corneum, whereas stratum granulosum and spinosum exhibit a typical honeycombed pattern. On the deeper sections (**D**–**F**), a dark, coiled tubular structure is visible (red asterisk). Moreover, bright cells around dermal papillae, which form edged dermal papillae (white asterisks), are seen at the entire level of the dermo–epidermal junction.

**Figure 4:**
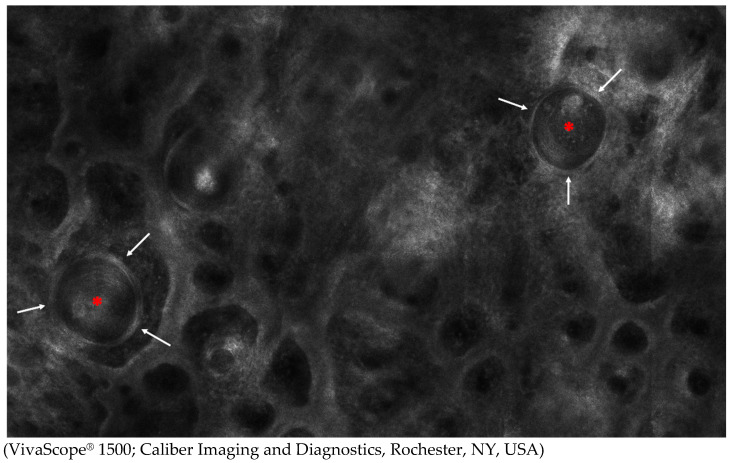
Reflectance confocal microscopy of syringoma. Epithelial cells (white arrows) forming ducts filled with a grey amorphous material (red asterisks) are visible in the dermis. Data on RCM features of syringoma is scarce, as only one report on RCM attributes of ES has been published so far. Jiménez et al [14]. reported RCM presentations of 2 syringomas located on the face and neck in patients diagnosed with ES; however, RCM images were not correlated with corresponding dermoscopy. Nevertheless, our RCM findings are in line with the observations of Jiménez et al. [14]. Similar to the Brazilian authors, we identified ducts surrounded by pigmented epithelial cells, some of which were filled with a grey amorphous material. Additionally, we identified acrosyringia in the epidermis in both of the presented instances, which seem to correspond to white pinpoint dots observed under dermoscopy. Although the utility of RCM in syringoma recognition is limited, as histopathological features of syringomas are confined to the upper dermis, identification of acrosyringia in the epidermis, along with ducts filled with amorphous material in the dermis, may narrow the differential diagnosis.

**Figure 5:**
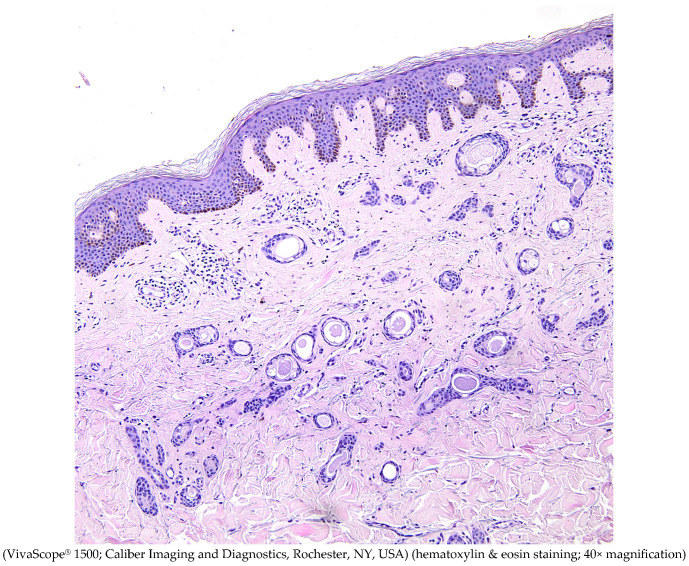
Syringoma displays multiple small ducts lined with cuboidal epithelial cells, some forming a characteristic ‘tadpole’ pattern, embedded in fibrous stroma within the dermis. Histology of syringoma typically reveals multiple small ducts and epithelial cords within the dermis, along with cystic eccrine ducts that often exhibit a characteristic comma-shaped tail [2,15].

## Data Availability

The original contributions presented in this study are included in the article. Further inquiries can be directed to the corresponding author.

## Supplementary Materials

The following supporting information can be downloaded at: https://www.mdpi.com/article/10.3390/diagnostics15010110/s1, Table S1: Dermoscopic and reflectance confocal microscopy (RCM) features of the entities that may mimic eruptive syringoma. References [16,17,18,19,20,21,22,23] are cited in the Supplementary Materials.

## References

[1] Hartmann K., Escribano L., Grattan C., Brockow K., Carter M.C., Alvarez-Twose I., Matito A., Broesby-Olsen S., Siebenhaar F., Lange M. (2016). Cutaneous Manifestations in Patients with Mastocytosis: Consensus Report of the European Competence Network on Mastocytosis; The American Academy of Allergy, Asthma & Immunology; And the European Academy of Allergology and Clinical Immunology. J. Allergy Clin. Immunol..

[2] Lei H., Wang Z., Ma X., Zhang Z., Feng Y., Zheng Y. (2023). Eruptive Syringomas: Summary of Ninety Cases and a Brief Literature Review. J. Cosmet. Dermatol..

[3] Papageorgiou M., Theodosiou G., Mandekou-Lefaki I. (2017). Eruptive Syringomas: Unresponsiveness to Oral Isotretinoin. Int. J. Dermatol..

[4] Marrogi A.J., Wick M.R., Dehner L.P. (1991). Benign Cutaneous Adnexal Tumors in Childhood and Young Adults, Excluding Pilomatrixoma: Review of 28 Cases and Literature. J. Cutan. Pathol..

[5] Huang Y.H., Chuang Y.H., Kuo T.T., Yang L.C., Hong H.S. (2003). Vulvar Syringoma: A Clinicopathologic and Immunohistologic Study of 18 Patients and Results of Treatment. J. Am. Acad. Dermatol..

[6] Williams K., Shinkai K. (2016). Evaluation and Management of the Patient with Multiple Syringomas: A Systematic Review of the Literature. J. Am. Acad. Dermatol..

[7] Friedman S.J., Butler D.F. (1987). Syringoma Presenting as Milia. J. Am. Acad. Dermatol..

[8] Hassab-El-Naby H.M.M., Nouh A.H. (2023). Syringomatous Dermatitis: A Myth or an Existing Entity?. Arch. Dermatol. Res..

[9] Wallace M.L., Smoller B.R. (1995). Progesterone Receptor Positivity Supports Hormonal Control of Syringomas. J. Cutan. Pathol..

[10] Bolognia J., Schaffer J.V., Cerroni L., Callen J.P. (2025). Dermatology.

[11] Botsali A., Caliskan E., Coskun A., Tunca M. (2020). Eruptive Syringoma: Two Cases with Dermoscopic Features. Skin. Appendage Disord..

[12] Sakiyama M., Maeda M., Fujimoto N., Satoh T. (2014). Eruptive Syringoma Localized in Intertriginous Areas. J. Dtsch. Dermatol. Ges..

[13] Hayashi Y., Tanaka M., Nakajima S., Ozeki M., Inoue T., Ishizaki S., Fujibayashi M. (2011). Unilateral Linear Syringoma in a Japanese Female: Dermoscopic Differentiation from Lichen Planus Linearis. Dermatol. Rep..

[14] Jiménez M.R., Rocchetto H., Ferreira P.S., Sangueza M., Lourenço S.V., Nico M.M.S. (2017). Evaluation of Syringomas by in Vivo Reflectance Confocal Microscopy: A Report of Two Cases. Am. J. Dermatopathol..

[15] Aleissa M., Aljarbou O., Aljasser M.I. (2021). Dermoscopy of Eruptive Syringoma. Skin Appendage Disord..

[16] Yan Q., Wang X. (2021). Dermoscopic and reflectance confocal microscopy features of eruptive xanthoma. Skin Res. Technol..

[17] Pogorzelska-Antkowiak A., Corneli P., Zalaudek I., Szepietowski J.C., Agozzino M. (2021). Characteristics of granuloma annulare in reflectance confocal microscopy. Dermatol. Ther..

[18] Zhang G., Chen J., Liu X., Wang X. (2020). Concordance of reflectance confocal microscopy with histopathology in the diagnosis of mastocytosis: A prospective study. Skin Res. Technol..

[19] Slawinska M., Kaszuba A., Lange M., Nowicki R.J., Sobjanek M., Errichetti E. (2022). Dermoscopic Features of Different Forms of Cutaneous Mastocytosis: A Systematic Review. J. Clin. Med..

[20] Lacarrubba F., Ardigo M., Di Stefani A., Verzi A.E., Micali G. (2018). Dermatoscopy and Reflectance Confocal Microscopy Correlations in Nonmelanocytic Disorders. Dermatol. Clin..

[21] Chen L., Wang Y., Gao X., Qin B., Lian J., Ren M., Zhang W., Wei R., Li Q. (2022). In vivo evaluation of facial papule dermatoses with reflectance confocal microscopy in children. Skin Res. Technol..

[22] Chen L.X., Wang Y., Qin B., Gao X.B., Li Q.F. (2021). Features of hypopigmented verruca plana in reflectance confocal microscopy and comparative analysis of hypopigmented and classic verruca plana in children. Skin Res. Technol..

[23] Panchaprateep R., Tanus A., Tosti A. (2015). Clinical, dermoscopic, and histopathologic features of body hair disorders. J. Am. Acad. Dermatol..

