# Supplementary material for: Eruptive Syringoma—Clinical, Dermoscopic, and Reflectance Confocal Microscopy Features
Source: Diagnostics (Basel). 2025 Jan 4;15(1):110. doi: 10.3390/diagnostics15010110 (PMC11719844; doi:10.3390/diagnostics15010110)
Supplement: Supplementary file 1 [file diagnostics-15-00110-s001.zip › diagnostics-3372908-supplementary.pdf]

Supplementary Table S1. Dermoscopic and reflectance confocal microscopy (RCM) features of the entities that may mimic eruptive syringoma

Diagnosis	Dermoscopy	Reflectance confocal microscopy			
		Epidermis	Dermo-epidermal junction	Dermis	Vascular structures
Eruptive xanthomas [1]	yellow-orange structureless areas with arboriform telangiectasias, surrounded by an erythematous-brown halo	-	roundish, discoid cells with peripheral hyper-refractile cytoplasm	-	-
Disseminated granuloma annulare [2]	yellowish-orange structureless areas or pinkish structureless areas with dotted vessels	• typical honeycombed pattern<br>• debris of keratinocytes and inflammatory cells<br>• clusters of inflammatory cells in intrapapillary space	bright basal cells in some areas with no clear borders due to inflammation		tops of papillae with dilated vessels in the spinous layer
Urticaria pigmentosa/maculopapular skin mastocytosis (MC)* [3,4]	brown structureless areas, brown lines arranged in a network, and linear vessels distributed in a reticular pattern	honeycomb-like pattern with widening of the intercellular space	-	the superficial dermis rich and plump wit edema; mast cell nuclei are hardly visible, and	capillary dilatation in dermal papilla

				cell boundary are unclear	
Lichen planus [5]	• whitish lines in a reticular, arboriform, annular, globular, or homogeneous arrangement<br>• linear vessels with radial distribution at the periphery of the whitish lines	hypergranulosis	diffuse dermoepidermal junction obscuration (sheets of inflammatory cells at the level of the interface between the epidermis and the upper dermis)	dilated dermal vessels	-
Flat warts [6,7]	-	• epidermis thickening<br>• petal-like structures in the stratum granulosum and the stratum spinosum	hypo-/hyperrefractive dermal papillary rings	dilated blood vessels and inflammatory cells	-
Eruptive vellus hair cysts [8]	• circular structure, with central yellowish to whitish papule<br>• erythematous brownish halo	Not reported			

* In a study by Zhang et al. [3] RCM features of 200 cases of cutaneous mastocytosis were analyzed together including 170 cases of urticaria pigmentosa/maculopapular skin MC, 18 cases of cutaneous mastocytoma, and 12 cases of diffuse cutaneous MC.

References

1. Yan, Q.; Wang, X. Dermoscopic and reflectance confocal microscopy features of eruptive xanthoma. *Skin Res Technol* **2021**, *27*, 639-640, doi:10.1111/srt.12988.
2. Pogorzelska-Antkowiak, A.; Corneli, P.; Zalaudek, I.; Szepietowski, J.C.; Agozzino, M. Characteristics of granuloma annulare in reflectance confocal microscopy. *Dermatol Ther* **2021**, *34*, e15021, doi:10.1111/dth.15021.
3. Zhang, G.; Chen, J.; Liu, X.; Wang, X. Concordance of reflectance confocal microscopy with histopathology in the diagnosis of mastocytosis: A prospective study. *Skin Res Technol* **2020**, *26*, 319-321, doi:10.1111/srt.12779.
4. Slawinska, M.; Kaszuba, A.; Lange, M.; Nowicki, R.J.; Sobjanek, M.; Errichetti, E. Dermoscopic Features of Different Forms of Cutaneous Mastocytosis: A Systematic Review. *J Clin Med* **2022**, *11*, doi:10.3390/jcm11164649.
5. Lacarrubba, F.; Ardigo, M.; Di Stefani, A.; Verzi, A.E.; Micali, G. Dermatoscopy and Reflectance Confocal Microscopy Correlations in Nonmelanocytic Disorders. *Dermatol Clin* **2018**, *36*, 487-501, doi:10.1016/j.det.2018.05.015.
6. Chen, L.; Wang, Y.; Gao, X.; Qin, B.; Lian, J.; Ren, M.; Zhang, W.; Wei, R.; Li, Q. In vivo evaluation of facial papule dermatoses with reflectance confocal microscopy in children. *Skin Res Technol* **2022**, *28*, 703-707, doi:10.1111/srt.13170.
7. Chen, L.X.; Wang, Y.; Qin, B.; Gao, X.B.; Li, Q.F. Features of hypopigmented verruca plana in reflectance confocal microscopy and comparative analysis of hypopigmented and classic verruca plana in children. *Skin Res Technol* **2021**, *27*, 993-996, doi:10.1111/srt.13034.
8. Panchaprateep, R.; Tanus, A.; Tosti, A. Clinical, dermoscopic, and histopathologic features of body hair disorders. *J Am Acad Dermatol* **2015**, *72*, 890-900, doi:10.1016/j.jaad.2015.01.024.
